# Open Letter upon the Prevention of Yellow Fever by Inoculation

**Published:** 1894-01

**Authors:** 


### Editorial.

### OPEN LETTER UPON THE PREVENTION OF YELLOW FEVER BY INOCULATION.

The accompanying letter and communication explain themselves, except to state that my letter appeared in the *Journal of Am. Med. Ass.*, Jan. 6, '94.

CHICAGO, ILL., Dec. 3rd, 1893.

*Dr. J. McF. Gaston, Atlanta, Ga.*

DEAR DOCTOR: Can't you send us a contribution for the Journal for December. Please send something on one of your favorite topics, and oblige. Faithfully yours,

JOHN B. HAMILTON, Editor.

---

ATLANTA, GA., Dec. 15th, 1893.

MR. EDITOR: As your are aware I have taken great interest in the matter of preventive inoculation against yellow fever since the publication of the experience of Dr. Domingus Freire appeared in Brazil, more than ten years ago. My efforts were first directed to enlisting the influence of Dr. Joseph Holt, of New Orleens, in support of a thorough investigation of this subject, under the impression that the people of that city ought to feel an interest in the results of such an inquiry.

As a sequel of the agitation of this question by him before the American Public Health Association and through the public prints, it was deemed proper by the United States Government to send Dr. G. M. Sternberg, U. S. A., to Rio de Janiero, to observe the working of yellow fever inoculation, and report upon the same.

Being impressed with the great difficulties likely to be encountered by one individual in getting a proper understanding of the facts presented in connection with the use of inoculation in a foreign land, I sought to secure the co-opeartion of others in this undertaking.

Resolutions were adopted at the meeting of the American Medical Association at Chicago, in 1887, upon a motion by me, requesting the President of the United States to send two other competent members of the medical profession to assist in getting the data requisite for a proper comprehension of what had been accomplished by Dr. Freire.

Upon your motion, this action was rescinded, after a vote of a large majority of the association against a motion for reconsideration. This was clearly without the sanction of parliamentary usage and yet availed to arrest any further steps in that direction.

The unfavorable verdict of the special commissioner is well known to all who are conversant with this matter, and was such as might have been expected from his mode of conducting the investigation.

Only upon one occasion, since that time, have I made any effort to bring this subject to the attention of the profession, or the people of this country.

But had I been present at the meeting of the American Medical Association, at Nashville, when the President in his annual address undertook to commiserate the short comings of Freire, I should have met him with facts.

In like manner, your comments upon yellow fever inoculation in your opening address, as chairman of the section of State Medicine, would most assuredly have received my attention, appreciating fully the complimentary terms in which you were pleased to refer to me. I suppose the snap judgment of the committee who passed upon the claims of yellow fever inoculation at that time may have been considered final by the section ; and that you may have written its epitaph, with *requiescat en pace* inscribed upon the mausoleum. But the stone placed upon the tomb and the seals affixed are destinəd earlier or later to be removed, and it will be verified, that "truth crushed to earth will rise again."

I am in possession of facts confirmatory of all that has been alleged in favor of the results of inoculation against yellow fever, as put in practice by Domingos Freire, in Brazil, and expect to present them before the medical profession at an early day.

It has not been from any diminution of my faith in the efficacy of inoculation in modifying materially, or preventing entirely, the access of yellow fever, that I have kept quiet during the past five years in regard to it. My time and attention have been too much occupied latterly with matters which were of more importance to me personally and professionally, than the attempt to convince people against their will of the reality of the exemption secured by yellow fever inoculation. But some of the problems, which I have been working out, are now solved ; and I am disposed to take up again the investigation of the claims of inoculation to the adoption of our people in the south.

I was pleased to learn from Dr. Holt during my attendance at the meeting of the Southern Surgical and Gynecological Association in New Orleans, that he has not despaired of realizing the benefits of inoculation as a prophylactic against yellow fever. Notwithstanding the diatribe against Freire's methods in an editorial of the New Orleans Medical Journal of January, 1886, and the soft impeachment against Dr. Holt and myself as having acted precipitately, and without due deliberation and consideration in advocating his claims to recognition, we are very far from giving up the fight in behalf of inoculation as a phophylactic measure against yellow fever.

The attitude of experts in regard to the immunity afforded by an attack of yellow fever, comes to the support of what has been demonstrated by the very small fatality of persons not acclimated, who have been inoculated in Brazil. I am aware that bacteriologists of repute claim that no such microbe as the cryptococcus xanthogenicus, which Freire has described, can be discovered by a scientific investigation. But let it denied that such a representative element exists in the form he has presented it, this fact does not set aside the positive results obtained with his attenuated virus as a preventive against this dreaded disease.

I am not aware that the microbe of small-pox or that of the cow-pox has been definitely determined by bacteriologists. Dr. Eugene Foster in his article (Ref. H., Med. Sciences, p. 480) says: "We have no satisfactory proof that either vegetable germs or bacteria, constitute the essential elements of the disease." Yet no scientific investigator at present hesitates to accept the plan of vaccination introduced by Jenner for preventing or modifying materially, the access of variola.

Neither has the microbe of rabies been ascertained with a certainty in the investigations of Pasteur, but he continues to record the prophylactic virtues of the rabic virus obtained from the dessiccated medullæ of infected rabbits. His last report of the treatment of persons bitten by rabid animals in the Pasteur Institute at Paris, shows a fatality of less than ½ of 1 per cent., and the reports from the Chicago Institute and the New York Institute, indicate like favorable results, in this class of cases. It is evident, therefore, that past experience in this prophylactic mode of dealing with such disorders, has proved eminently satisfactory without a recognition of microbiology in using an attenuated virus. Call it empiricial, but the efficacy cannot be questioned.

I have an abiding conviction that you would magnanimously admit the claims of this great prophylactic measure for the protection of our Southern ports, if you could see this matter in its true light. Your energetic administratron of the sanitary work of the marine service in former years, must have impressed our people with your discretion and zeal in adopting efficient measures against the spread of yellow fever. Should your mind be directed to a thorough comprehension of the details of the practical working of yellow fever inoculation, disconnected with any scientific investigation of the microbial element, I should expect you to be so impressed with its benefits, that at least you should be disposed to test this process in places where yellow fever may occur in future years. If this is done, I shall yet realize my fondest hopes in the adoption of this process amongst us. Yours sincerely,

J. MCFADDEN GASTON.

